# Analysis of mandibular trabecular bone by fractal analysis in children born with oral cleft

**DOI:** 10.1186/s12903-026-08161-5

**Published:** 2026-03-24

**Authors:** Caio Luiz Bitencourt Reis, Bianca Lopes Cavalcante-Leão, Gabriela Fonseca-Souza, Luisa Helena Batista, Mirian Aiko Nakane Matsumoto, Rafaela Scariot, Daniela Adacheski, Nikolaos Daratsianos, Svenja Beisel-Memmert, Juliana Feltrin-Souza, Erika Calvano Küchler

**Affiliations:** 1 School of Dentistry, Federal University of Alfenas, Alfenas, Minas Gerais Brazil; 2 School of Dentistry, Positivo University, Curitiba, Paraná Brazil; 3 School of Dentistry, Federal University of Paraná, Curitiba, Paraná Brazil; 4 Department of Pediatric Dentistry, School of Dentistry of Ribeirão Preto, University of São Paulo, Ribeirão Preto, Brazil; 5 Department of Orthodontics, Medical Faculty, University Hospital Bonn, Welschnonnenstraße 17, Bonn, 53111 Germany

**Keywords:** Oral cleft, Bone, Mandible, Condyle, Birth defect, Congenital anomaly

## Abstract

**Introduction:**

This study aimed to investigate if the mandibular trabecular bone structure of children and teenagers with cleft lip and/or cleft palate (CL/P) differs from non-cleft children using fractal analysis.

**Methods:**

Children aged between 5 and 15 years with CL/P (cleft group) and a control group were recruited. Syndromic cases of CL/P and isolated cleft palate (CP) were excluded. To obtain the fractal dimension (FD) of the mandibular bone, regions of interest (ROI) were first delineated within the condyle and body of the mandible on panoramic radiographs. Subsequently, the FD was calculated using the box-counting algorithm on these defined ROIs. The results were adjusted by sex and age in a generalized linear model (GLM) with alpha error tolerance of 5%.

**Results:**

A total of 205 patients were included. One hundred patients were allocated into the control group and 105 into the cleft group (37 CL and 68 CLP). The fractal dimension (FD) was statistically higher in the control group compared to the study group for both regions of interest (ROIs) (*p* < 0.001). In the GLMs, cleft was associated with low FD in the condyle (Beta=-0.132; *p* < 0.001). In the body of the mandible, cleft was also associated with decreased FD values (Beta=-0.154; *p* < 0.001). CLP presented decreased FD values in the condyle (Beta=-0.016; *p* = 0.004).

**Conclusion:**

Children with CL/P had lower FD values in the mandible compared to the control children, which may indicate that patients born with CL/P present a less complex bone structure.

**Supplementary Information:**

The online version contains supplementary material available at 10.1186/s12903-026-08161-5.

## Introduction

Craniofacial birth defects rank as one of the most frequent congenital anomalies observed in newborns. Oral clefts represent the most common congenital deformity of the craniofacial complex [1]. Oral clefts include cleft lip (CL), cleft lip and palate (CLP), and cleft palate only (CP). CL and CLP are often grouped together as a single condition due to their epidemiological, embryological, and genetic similarities [2]. CP involves a normal initial formation of the lips and alveolar ridge, but is characterized by the failure of the palatine processes to successfully merge at the midline or with the nasal septum, leaving a direct communication between the oral and nasal cavities [2]. Cleft lip with or without cleft palate (CL/P) occur either in combination with one or more additional anomalies as part of a syndrome spectrum (syndromic cleft) or as an isolate trait (non-syndromic cleft). Non-syndromic CL/P is more prevalent, accounting for approximately 70% of cases [3]. A Surveillance data collected from birth defect registries around the world [4] shows that the prevalence of CLP varied substantially, ranging from 1.26 to 10.37 per 10,000 births. Among the epidemiological differences between CL/P and isolated CP is the well-documented sex preference of oral cleft subphenotypes, with male predilection to CL/P and female predilection to CP, in which the CL/P sex ratio is approximately 2:1 (male: female) [5].

The development of the lip and palate during embryonic craniofacial development involves disturbance of various embryonic tissues, such as epithelial and bone tissues. Disruptions in specific genes and proteins responsible for regulating cell migration, growth, differentiation, and apoptosis in these tissues can lead to the development of CL/P [6]. Genetic studies of CL/P extend back centuries, and the results show that many genes seem to influence the risk of this condition. There is a vast diversity in the functions of the cleft-associated genes, and interestingly, several genes, such as *FGFR1*, and *WNT10A*, play a key role in bone development [7, 8], suggesting a sharing molecular signals for CL/P and bone development.

Due to the fact that bone-related genes and proteins have been associated with CL/P, it is possible that CL/P patients present some differences in the structural patterns of bone tissue. In fact, some recent studies report morphometric distinct characteristics of bone structures in CL/P patients [9, 10]. Fractal analysis is a method for the quantitative morphometric evaluation of bone [11]. This method identifies intricate structural patterns in trabecular bone and quantifies its complexity through a metric known as fractal dimension (FD). Several recent studies suggested that fractal analysis can be useful in evaluating the mandible trabecular bone through panoramic radiographs, allowing for the assessment of bone quality and structural integrity [12–16]. Although many studies have been applying fractal analysis to investigate mandibular trabecular bone in variety of conditions, to the best of our knowledge, no previous studies have evaluated mandibular trabecular bone in children with CL/P via fractal analysis. Therefore, the aim of this study was to investigate if mandibular trabecular bone structure of children and teenagers with CL/P differs from non-cleft children by using the fractal analysis.

## Materials and methods

This cross-sectional study was reported according to STROBE Statement [17]. This study followed the Declaration of Helsinki and was approved by the Committee for Ethics in Research with Humans of the Department of the State Health Department of Paraná (protocol number 5.100.185) and by the Federal University of Paraná (protocol number 3.752.172). The researchers invited the minor patients and their legal guardians and presented a document that clarified the study’s details. The legal guardians who accepted that the minor take part in the study expressed their consent by providing a handwritten signature. Minor patients received a document suitable for their age and signed it with a handwritten signature. All patients were competent in reading and writing.

### Setting

The sample was selected through convenience sampling method from September 2021 until October 2023. The participants born between 2007 and 2018 were recruited from a center for craniofacial anomalies treatment (Comprehensive Care Center for Cleft Lip and Palate) and in the pediatric dental clinic at the Federal University of Paraná. Both centers are located in Curitiba. Brazil.

### Participants

This study recruited children without CL/P (control group), as well as children with CL/P (cleft group). The participants from control and cleft groups were both genders and aged between 5 and 15 years. The diagnosis of children in the cleft group was conducted in accordance with the criteria set by Martinelli et al. [18]. The specific type of cleft was determined thorough clinical examination and a review of medical records. The cleft group included cases of CL and CLP. Cases of isolated cleft palate (CP) were excluded due to their distinct congenital malformation. CP has a different embryologic origin and genetic distinct component compared to CL/P [19]. Additionally, children with other syndromes or craniofacial anomalies were not included in the study.

### Variables and data sources

A Region of Interest (ROI) is a specific area within an image designated for a particular analysis. FD were obtained from specific regions of interest (ROI) in the mandible using panoramic radiographs obtained from the participant’s medical records. This measurement quantifies the complexity and irregularity of complex structures that cannot be adequately described by traditional Euclidean analysis, such as the microarchitecture of bone. FD is particularly studied in osteoporosis research serving as a predictive indicator of fracture risk. FD is calculated to generate a single numerical value that summarizes the complexity of bone structure. In the context of 2D images, values that are close to 0 suggest a loss of complexity in bone tissue, indicating less interconnected trabeculae and a possible deterioration of the overall structure. While, values near 2 indicate a more intricate and well-connected trabecular network reflecting healthier bone architecture [12–16].

**Fig. 1:**
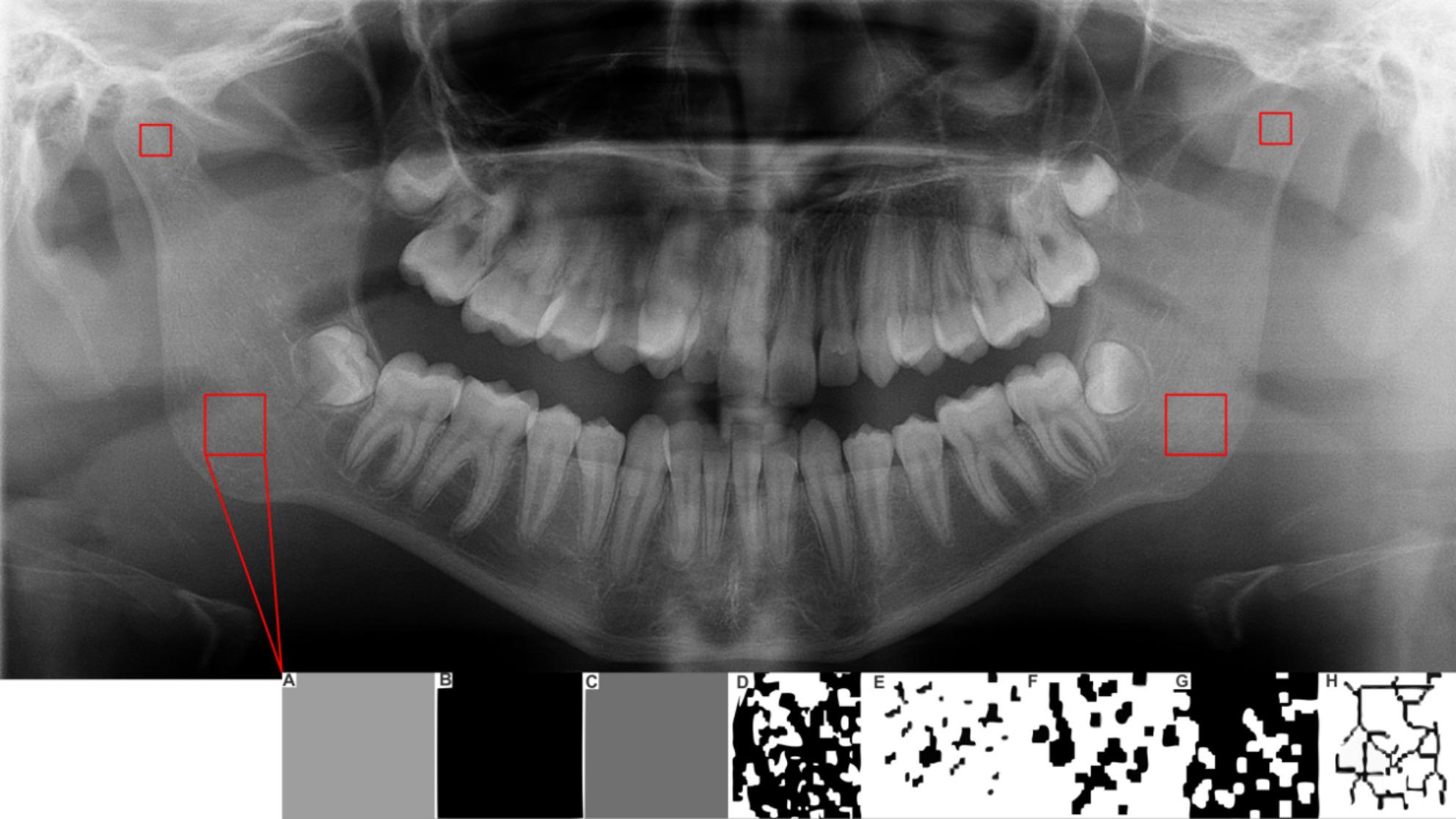
Representation of ROIs and the process to FD evaluation. Legend: The ROI of the condyle of the mandible was determined by a square form with 50 × 50 pixels placed in the geometric center of the head of the condyle. The ROI of body of the mandible was determined by a square form ROI with 100 × 100 pixels placed in the middle of the trabecular bone area between the angle of the mandible and the inferior cortical border of the mandible canal. **A** to **C** represents the Gaussian filter application. **D** to **G** represents the binarization process. **H** represents the skeletonized images

All panoramic radiographs from the included children presented high-quality requirements (i.e. without artefact and/or distortions) and were examined digitally in a darkroom by a single trained examiner. The TIF (Tagged Image File) file of radiography were standardized in width and height of 2976 × 1536 pixels. The ROI measuring was selected from the right and left side of the mandible as demonstrated in Fig. 1.

FD assessment was performed using the open source software Image J version 1.53 (National Institutes of Health). The images underwent a series of processing steps, starting with Gaussian Blur applied in two dimensions utilizing a sigma of 2.0. Following this, the images were binarized focusing on pixels with grey values ranging from 177 to 255, which were then subjected to skeletonization. The plugin BoneJ version 1.4.3 [20] was used to calculate the FD through the box-counting algorithm.

Additionally, age, sex and the type of cleft lip were also explored in this study.

### Bias

To avoid performance bias, inter- and intra-examiner tests were performed. An examiner (LHB) was trained and calibrated by an experienced researcher (CLBR). Both researchers blinded for sex and age accessed the ROI’s from panoramic radiographs of 15 patients who were not included in this study. The reliability of their assessments was measured using the intraclass correlation coefficient (ICC), which indicated a strong inter-examiner reliability of 0.85. After a period of 15 days, LHB re-evaluated the same 15 panoramic radiographs to assess intra-examiner reliability (ICC = 0.83). The examiner (LHB) limited the assessments to 15 panoramic radiographs per day over a period of at least 60 min.

To reduce information bias, an automated command script was developed in ImageJ to standardize all analytical steps, ensuring procedural consistency and minimizing the likelihood of human error during data processing. To avoid confounding bias, a multivariate analysis was performed.

### Study size

The sample size was calculated using the G*Power software (Version 3.1.9.7, University of Kiel, Germany). We applied the t-test for comparing means between two independent groups as the statistical model. The expected effect size was based on the results reported by Kurt et al. [21], who identified a Cohen’s d of 0.42. Assuming an alpha of 0.05 and a statistical power of 80%, the analysis indicated that at least 174 participants would be necessary to adequately power the study.

### Statistical methods

Children without CL/P were allocated into the “control” group and CL/P into the “case” group. CL/P children were also stratified by the cleft type (CL or CLP).

Age was handled as a quantitative variable. The Shapiro-Wilk test shows a normal distribution of data (*p* > 0.05) and the mean age with standard deviation (SD) were calculated. Age was compared between studied groups by Student’s T test without correction. The distribution of sex between groups was analyzed by Pearson’s Chi-square test without correction.

Due to the interdependence of the DF data (i.e. multiple ROIs from the same patient) statistical analysis was adjusted to accommodate multiple observations per participant. We used the “Complex samples” module in IBM SPSS for Windows version 25.0 (IBM. Chicago. IL. USA). This module considers the elements of complex sampling designs, such as strata (i.e. two ROIs at right and left sides) and clusters (i.e. participants) and apply appropriate statistical techniques to account multiple observations per participant to obtain accurate parameter estimates and variances. Briefly, analysis of variance (ANOVA) was firstly applied taking into account the sample design. including equal probability, and PPS (probability proportionate to size) methods, with or without sampling procedures. The mean and standard error (SE) of DF were calculated and compared between the groups by a hypothesis test (t-Student test) with adjustment for multiple comparisons (sequential Bonferroni). After the results were adjusted by sex and age in the generalized linear model (GLM), with alpha error tolerance of 5%.

## Results

Two hundred twenty-two (*n* = 222) patients were recruited. Twelve patients were excluded due to the involvement of cleft only in the palate. Five patients were excluded due to distortion in ROI’s. Finally, 205 patients were included. A total of 105 (53.4%) were allocated into the cleft group, and 100 (46.6%) allocated into the control group. In the cleft group, 37 (33.2%) were patients with CL, and 68 (64.8%) were patients with CLP.

Table 1 shows the characteristics of the sample according to the groups. The mean age was statistically different between CL and CLP groups (*p* = 0.030), but the cleft group was not different to the control group (*p* > 0.05). The distribution between sex were not different between groups (*p* > 0.05).

**Table 1:** Characteristics of the studied sample

Variables	Total	Control Group	Cleft Groups		
			All clefts	CL	CLP
Total (*n* and %)	205 (100)	100 (46.6)	105 (53.4)	37 (33.2)	68 (64.8)
Age (mean and SD)	8.76 (2.02)	8.50 (1.99)	9.00 (2.03)	8.68 (2.25)*	9.18 (1.90)*
Girls (*n* and %)	91 (44.4)	50 (50.0)	41 (39.0)	14 (27.0)	27 (29.7)
Boys (*n* and %)	114 (55.6)	50 (50.0)	64 (61.0)	23 (73.0)	41 (60.3)

SD means Standard Deviation. sex was compared between groups with Pearson’s chi-square test without correction. Age was compared between groups with Student’s t test

*Indicate statistical difference (*p* = 0.030)

FD was compared between groups and sex (Table 2). The FD was statistically greater in control the group compared to the cleft group, including the stratified cleft groups (CL and CLP) for both ROI’s. There was no statistical significance when the cleft group was stratified between boys and girls to both ROIs. When CL and CLP groups were compared, there was no statistical difference to both ROIs.

**Table 2:** Comparison of FD between studied groups and sex

Groups		ROI Condyle		ROI Mandibular Angle	
		Mean	SE	Mean	SE
Total		1.17	0.01	1.14	0.01
*Control Group*		1.24	0.02	1.22	0.01
	Girls	1.29	0.03	1.22	0.02
	Boys	1.20	0.03	1.23	0.02
*Cleft Group*		1.08	0.02	1.06	0.01
	CL	1.13	0.02	1.05	0.02
	CLP	1.10	0.01	1.08	0.01
	Girls	1.10	0.01	1.07	0.01
	Boys	1.11	0.02	1.08	0.01
*P*-values	Control group vs. Cleft group	<0.001		<0.001	
	Control group vs. CL group	<0.001		<0.001	
	Control group vs. CLP group	<0.001		<0.001	
	CL vs. CLP	0.203		0.211	
	Boys with cleft vs. Girls with cleft	0.103		0.394	

SE means standard error. The comparisons were made with ANOVA, followed by Student’s T test with Sequential Bonferroni’s adjust accounted for multiple observation per participant.

In generalized linear model, cleft was statistically associated with decrease of FD in the condyle (Beta = -0.132; *p* < 0.001), even when interacted with age (Beta − 0.015; *p* = 0.010) and sex (girls: Beta = -0.017; *p* = 0.003 / boys: Beta = -0.013; *p* = 0.034). In the body of the mandible, cleft was also associated with decrease of FD (Beta= -0.154; *p* < 0.001). However, when the interaction with age and sex was considered, this effect was no longer observed for the mandibular body (*p* > 0.05).

The stratified cleft groups were also evaluated in GLM (Table 3): when interacted with age, CLP was associated with decrease of FD in the condyle (Beta= -0.016; *p* = 0.004), but not with FD in the body of the mandible. Isolated CL was not associated with FD in both studied regions.

**Table 3:** Multivariate analysis per models

Models	ROI Condyle				ROI Mandibular Angle			
	Beta	SE	T value	*p*-value	Beta	SE	T value	*p*-value
Cleft	-0.132	0.02	-6.64	< 0.001	-0.154	0.01	-8.89	< 0.001
Age	-0.012	0.01	-2.18	0.030	-0.003	0.01	-0.594	0.553
Sex	0.004	0.02	0.169	0.866	0.003	0.02	0.142	0.887
Cleft * age	-0.015	0.01	-2.58	0.010	-0.006	0.01	-1.48	0.139
Cleft * age * sex								
Girl	-0.017	0.01	-2.98	0.003	-0.007	0.01	-1.68	0.093
Boy	-0.013	0.01	-2.12	0.034	-0.006	0.01	-1.25	0.209
CL * age	-0.012	0.01	-1.73	0.083	-0.009	0.01	-1.88	0.060
CLP * age	-0.016	0.01	-2.88	0.004	-0.006	0.01	-1.3	0.194

Each line refers a one model, separately. SE means Standard Error. FD was the dependent variable. The test was executed by generalized linear models accounted for multiple observation per participant

## Discussion

The multifactorial nature of nonsyndromic CL/P, and its complex interplay between environmental, developmental, and genetic factors, reflects in the clinical heterogeneity of the CL/P. In fact, evidence from epidemiological population-based studies shows that patients diagnosed with CL/P have high risk for other conditions, such as congenital heart disease [22], and dental anomalies [23]. Recently, disturbances in bone development were also associated with CL/P: patients diagnosed with CL/P present variations in the sella turcica phenotype [24, 25], in the mandibular cortical bone thickness [9], and in the upper cervical spine morphology [10]. Therefore, we hypothesize that mandibular trabecular bone structure in CL/P patients could also vary. In our study, we observed that children with CL/P presented a decrease of FD in the condyle and in the body of the mandible.

Our results bring some interesting hypotheses about the shared pathways for cleft development and bone formation. Genetic variants that influence the lip, and palate morphology have pleiotropic effects non-isolated to these structures [26], and also may impact the mandibular bone. A previous study [9] reported irregular bone micro-structure in cortical regions of the mandible in CL/P patients. In our study, we evaluated the trabecular structure of the mandible condyle and body, the mainly mandible growth regions. We quantify the structural quality of these regions by fractal analysis in panoramic radiographs. The low FD values of CL/P patients in these regions indicates a trabecula more porous and less organized. In fact, a systematic review pointed out that low FD values in regions of thmandible are associated with low bone mineral density (BMD) [27].

The facial bone quality assessment in patients with CL/P is highly important for clinical practice. The bone quality in these patients impacts both the craniofacial development and the long-term outcomes of treatment. Many patients born with CL/P require bone grafting, especially as they grow older and need more bone support for speech, breathing, and tooth eruption. Alveolar bone grafting provides essential structural stability to the dental arch and facilitates the proper eruption pathway of the permanent canine, since teeth adjacent to the cleft often exhibit altered eruption patterns, deficient bone support, or ectopic positioning. This procedure also contributes to improved periodontal health, supports future orthodontic tooth movement, and enhances overall maxillary continuity and function [28]. Therefore, the quality of the bone at the cleft site determines how well the graft will integrate and heal. Poor bone quality can lead to graft failure, increasing the risk of additional surgeries. Understanding bone quality is also important in surgery practice, once oral cleft surgeries often require the repositioning of bones to improve facial aesthetics and function. Although extrapolating the results from the mandible to the maxilla should be done with caution, our study raises some interesting points. The, reduced mandibular FD in children with CL/P does not necessarily imply an equivalent pattern in maxillary bone—particularly in the cleft region, however, it is possible that maxillary bone also follows a similar pattern. Future studies should perform fractal analysis in the maxilla of children with CL/P. It is important to emphasise that panoramic radiographs are less reliable for detailed evaluation of the maxilla near the cleft due to distortion and superimposition, but periapical radiographs or cone beam computed tomography -derived projections could allow more accurate and region-specific FD analysis.

Systematic review [29], epidemiological [30], and genetic study [31] performed in the past decades suggest that oral cleft and tooth agenesis share a similar genetic background [32]. Interestingly, a previous study that aimed to evaluate whether the mandibular trabecular bone structure of patients with tooth agenesis differs from the control group by using the fractal analysis, observed that mandibular trabecular bone quality of patients with one congenitally missing tooth was different from the healthy group, in which the fractal analysis values of the tooth agenesis group were lower than the control group [12].

While fractal analysis performed in panoramic radiographs can provide valuable insights into the structural complexity of bone, its limitations include issues related to image quality and geometric distortion due the inherent two-dimensional nature of the radiographic images. The challenges in defining and standardizing ROI must be taken into consideration. These factors can reduce the accuracy of fractal analysis in panoramic radiographs, especially when compared to more advanced imaging techniques, such as three–dimensional computed tomography. Future studies in different populations should be performed, once the evaluation in different settings are crucial for the generalizability of our research findings.

## Conclusion

Children with CL/P had lower fractal dimension values in the mandible compared to the control children, which may indicate that patients born with CL/P present a less complex bone structure. Understanding bone complexity in patients with oral cleft is crucial for etiological and clinical research. Future research should be conducted in diverse populations, including comprehensive evaluations of the maxilla, and should incorporate three-dimensional tomography analyses to achieve more accurate and detailed assessments.

## Supplementary Information

Supplementary Material 1.

## Data Availability

The datasets supporting the conclusions of this article is included within the article.
